# Improvements on the Stability and Vitamin Content of Acerola Juice Obtained by Ultrasonic Processing

**DOI:** 10.3390/foods7050068

**Published:** 2018-05-01

**Authors:** Valéria O. Santos, Sueli Rodrigues, Fabiano A. N. Fernandes

**Affiliations:** 1 Departamento de Engenharia Química, Universidade Federal do Ceara, Campus do Pici, Bloco 709, Fortaleza-CE 60440-900, Brazil; valeria.amora@gmail.com; 2 Departamento de Engenharia de Alimentos, Universidade Federal do Ceara, Campus do Pici, Bloco 858, Fortaleza-CE 60440-900, Brazil; sueli@ufc.br

**Keywords:** *Malpighia emarginata*, acerola, ultrasound, stability, nutritional quality

## Abstract

This work has examined the influence of ultrasonic processing on acerola juice and its influence in the stability of the juice and in the availability of vitamins B, C, E, and pro-vitamin A. The study has evaluated the changes in these quality parameters resulting from changes on ultrasonic power density, processing time and temperature. Ultrasound application increased the availability of pro-vitamin A and vitamins B_3_, B_5_, C and E in the juice by releasing them from the apoenzymes to which they are bound and by improving the homogeneity of the juice. The retention of the major vitamins in acerola juice (vitamins A and C) was higher when lower temperatures (10 to 20 °C) and mild ultrasound power density (2000 to 3000 W/L) were applied.

## 1. Introduction

An industrial process designed for fruit juice preservation needs to focus not only on the juice safety, but also on retaining its vitamin content and reducing the formation of undesirable breakdown products. If possible, it should also aim at increasing the bioavailability of vitamins and other bioactive compounds.

Thermal pasteurization ensures food safety and extended shelf-life preservation by denaturation of enzymes and microbial decontamination. However, the use of heat may affect the nutritive and sensorial quality of fruit juices negatively.

Among the non-thermal technologies, ultrasonic processing is effective against microorganism spoilage and undesired enzymatic effects (either alone or in combination with other preservation techniques). Several studies have shown that ultrasonic processing meets the five-log reduction requirement for contaminants in fruit juices [1,2,3,4].

The effect of ultrasonic processing on the nutritive quality of fruit juices has been addressed mainly on vitamin C and total phenolics [5,6,7]. Little is known regarding the effects of ultrasonic processing on pro-vitamin A, vitamin E and B vitamins.

The intestinal absorption of vitamins depends on the chemical form and physical state in which the vitamin exists within the food matrix. In fruits, the B vitamins occur as their coenzyme derivatives, usually associated with their protein apoenzyme. Vitamins that exist as chemically-bound complexes in the food matrix exhibit lower digestion and lower absorption efficiency compared to the free form of the vitamin [8].

Ultrasonic energy can break the bond between vitamins and their coenzymes. Thus, ultrasonic processing can generate the more bioavailable free-form of these vitamins. When apples were subjected to ultrasound-assisted air-drying, the content of free vitamins B_1_, B_2_, B_3_ and B_6_ increased [9]. Ultrasound-assisted air-dried tomatoes also presented an increase in the contents of vitamins B_1_, B_2_, B_3_, B_5_ and B_6_ [10].

Acerola is a common berry in South America and the Caribbean. It is used in the production of juices given its low sugar content and slight bitterness. Acerola is a good source of pro-vitamin A and vitamin C, and it also contains vitamins B_2_, B_3_, and B_5_ in lesser amounts [11].

In this work, acerola juice was subjected to ultrasonic processing, under the conditions usually applied in fruit juice preservation. The influence of its application on vitamins A, B_2_, B_3_, B_5_, C and E, as well as the cloud stability was evaluated.

## 2. Materials and Methods

### 2.1. Preparation of Samples

Acerola (*Malpighia emarginata*) was bought from a local producer (Fortaleza, Brazil) in the form of frozen pulp. The process used by the producer consists of pressing the seedless berries and freezing the pressed pulp without the incorporation of any additive.

The acerola juice was prepared mixing 100 g of acerola pulp with 100 mL of water (1:1 weight ratio). The juice was prepared using a household blender, and homogenization was attained in approximately 30 s. This preparation is like most ready-to-drink acerola juices.

### 2.2. Ultrasonic Processing and Experimental Design

Ultrasonic processing was carried out using an 18-kHz probe ultrasound (Unique Model USD500, Indaiatuba, Brazil) with 500 W of nominal power. The probe consisted of a 13-mm titanium probe, which was immersed 15 mm below the liquid surface. For each experiment, 100 mL of acerola juice were placed in a glass jacketed beaker (250 mL). The juice was subjected to ultrasonic application for 2.5, 5, 10 and 15 min.

A 2^3^ experimental design was carried out to evaluate the effects of ultrasound power density and process temperature on the vitamin content and the cloud stability of the juice. Three different levels of ultrasonic power densities were evaluated: 1000, 3000 and 5000 W/L (corresponding to 20%, 60% and 100% of the maximum power of the ultrasound equipment). Three levels of temperatures were evaluated: 10, 25 and 40 °C (corresponding to cold processing, room temperature processing and processing at mild temperature). The temperature was maintained constant by circulating refrigerated or heated water through the jacket of the glass jacketed beaker. The circulating water was cooled/heated using a thermostatized bath (Tecnal Model TE-184, Tecnal, Piracicaba, Brazil). All experiments were carried out in duplicate. The full experimental design is presented in Table 1.

A reference experiment without ultrasound application was carried out at each temperature to differentiate between the ultrasonic and the thermal effect.

### 2.3. Determination of Vitamins

To evaluate the influence of the process on the product quality, the relative content of vitamins B_2_, B_3_, B_5_, E and pro-vitamin A were determined based on the methods reviewed and compiled by Jedlicka and Klimes [12] and Rizzolo and Polesello [13] and described by Fernandes et al. [9]. The results were expressed as vitamin gain/loss using the fresh juice as a reference, as presented in Equation (1).

(1) $$\mathrm{Relative}\;\mathrm{amount}=\left(\frac{{ABS}_{Sample}}{ABS_{Reference}}\right)\times 100$$

The determinations were carried out at the wavelength of maximum absorbance of each vitamin, which was determined previously using pure vitamin standards purchased from Sigma-Aldrich (Sigma-Aldrich, St Louis, MO, USA).

For pro-vitamin A and vitamin E, 1 mL of juice was mixed with 6 mL of distilled water. Sodium hydroxide 0.5 M (1 mL) was added to the sample, which was heated for 30 min in a water bath at 70 °C. This protocol was done to saponify oils that may interfere with the determination of vitamins A and E (which are not saponified under these conditions). Hexane (5 mL) was added, and the mixture was vigorously stirred in a vortex for 1 min. The supernatant (hexane phase) containing the lipid-soluble vitamins (pro-vitamin A and vitamin E) was collected and analyzed spectrophotometrically at 215 (vitamin E) and 325 nm (pro-vitamin A) using hexane as the blank. Quartz cuvettes with a 10-mm optical path were used. All analyses were carried out in triplicate. Results were expressed as vitamin gain/loss using the vitamin content of the untreated juice as a reference (100%).

For the B vitamins, 1 mL of juice was mixed with 6 mL of distilled water. The vitamins were extracted adding sulfuric acid 0.25 M (1 mL) to the sample, which was heated for 30 min in a water bath at 70 °C. After cooling, the pH of the mixture was adjusted to pH 4.5 using a 0.5 M sodium hydroxide solution. The sample was centrifuged at 10,000 rpm (8400× *g*) for 10 min. The supernatant containing the water-soluble vitamins was collected and analyzed spectrophotometrically at 215 (vitamin B_5_), 254 (vitamin B_2_) and 265 nm (vitamin B_3_) using water as a blank. It was not possible to quantify the vitamin B6 content in acerola using the spectrophotometric method due to the presence of interferons. All analyses were carried out in triplicate and the results were expressed as vitamin gain/loss using the vitamin content of the untreated juice as a reference (100%).

Vitamin C content was determined using the oxalate method [14]. A sample of the juice (0.2 mL) was mixed with 1.5 mL of 0.005 mol/L sodium oxalate solution. This mixture was left standing for 5 min to extract the vitamin. Absorbance readings were made at 266 nm, using the 0.005 mol/L sodium oxalate solution as a blank. A calibration curve was made using l-ascorbic acid as a standard. Results were expressed as vitamin gain/loss using the vitamin C content of the untreated juice as a reference (100%).

### 2.4. Cloud Stability

The cloud stability was evaluated determining the cloud value of the juice. The cloud value was determined by UV-Vis analysis [15]. A sample of the juice (5 mL) was centrifuged for 10 min at 10,000 rpm (8400× *g*) (Sigma Model 3–16 KL centrifuge, Osterode am Harz, Germany), and the supernatant was analyzed. Absorbance readings were made at 660 nm in a UV-Vis spectrophotometer (Thermo Fisher, Waltham, USA). Three measurements were taken from each sample. The results were reported as the mean ± the standard deviation.

### 2.5. Statistical Analysis

The results were evaluated using the response surface methodology (analysis of perturbation of factors and ANOVA). The LSD (least significance difference) intervals (*p* < 0.05) were estimated. Statistical analysis was carried out using the software package Statistica v.13 (Tibco Software, Palo Alto, CA, USA).

## 3. Results and Discussion

### 3.1. Vitamin Content

**Figure 1:**
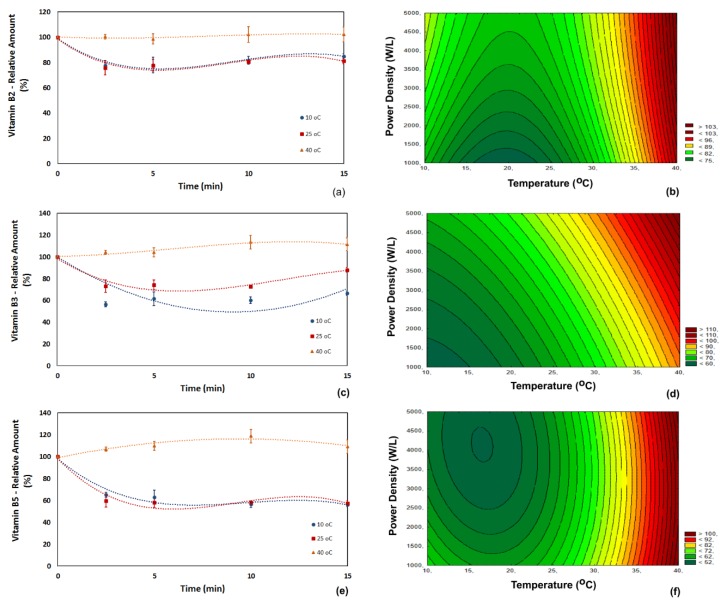
Influence of temperature, power density and processing time on the relative content of vitamins B in acerola juice. (**a**,**b**) Vitamin B_2_; (**c**,**d**) vitamin B_3_; (**e**,**f**) vitamin B_5_. The kinetics (**a**,**c**,**e**) was obtained applying a power density of 5000 W/L, and the response surface plots (**b**,**d**,**f**) were built using the data obtained at 15 min of ultrasound processing.

**Figure 2:**
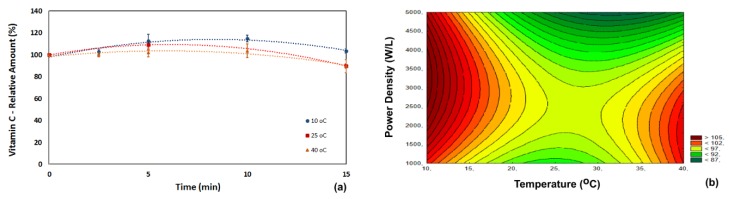
Influence of temperature, power density and processing time on the relative content of vitamin C in acerola juice. (**a**) Process carried out at 5000 W/L; (**b**) process carried out for 15 min.

**Figure 3:**
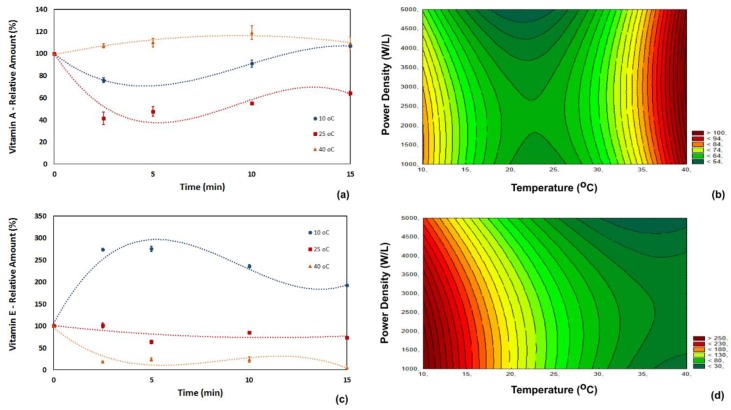
Influence of temperature, power density and processing time on the relative content of vitamins A (**a**,**b**) and E (**c**,**d**) in acerola juice.

The relative contents of vitamins in acerola juice subjected to ultrasonic processing are presented in Figure 1, Figure 2 and Figure 3, respectively for vitamins B (B_2_, B_3_ and B_5_), vitamin C and the lipid-soluble vitamins (A and E). The relative content was determined considering the vitamin content in the untreated acerola juice as 100%. The untreated acerola juice contained 0.08 mg/100 mL of vitamin B_2_, 0.40 mg/100 mL of vitamin B_3_, 0.031 mg/100 mL of vitamin B_5_, 1670 mg/100 mL of vitamin C, 760 UI/100 mL of pro-vitamin A and 0.02 mg/100 mL of vitamin E. The vitamin content of the acerola juice used in this study was within the range reported in the USDA Food Composition Database [11].

The vitamin B_2_ content decreased during ultrasonic processing at low and room temperature. Part of the vitamin B_2_ (riboflavin) in juices is found in its free-form and part is bound tightly to an apoenzyme [8]. Ultrasound application was not able to break the bond between the vitamin and the apoenzyme, changing it to its free and bioavailable form. As an overall trend for the process, the retention of vitamin B_2_ was higher at the highest temperature tested (an average of 100% at 40 °C against 80% at 10 °C). The degradation of vitamin B_2_ was mostly caused by ultrasonic processing rather than by the thermal effect because vitamin B_2_ is a thermally-stable vitamin. The effect of ultrasound was not significant at a high temperature (40 °C), and no significant change in vitamin B_2_ content was observed at this temperature. Higher degradation was observed at low temperatures because cavitation caused by ultrasound is more intense at low temperatures than at high temperatures [16] (Figure 1a,b).

The analysis of perturbation of factors (Table 2) showed that the primary factor influencing the changes in vitamin B_2_ content was the process temperature (*p* < 0.01) followed by the ultrasonic power density (*p* < 0.05), corroborating with the overall trends observed for this vitamin. The retention of vitamin B_2_ could be correlated with Equation (1) (*R*^2^ = 0.98).

(2) $$\mathrm{Retention}\;\mathrm{of}\;\mathrm{Vit}\;B_{2}\;\left(\%\right)=93.308-2.452T+0.063T^{2}+0.003P$$

The vitamin B_3_ (niacin) content presented a similar trend (Figure 1c,d). The retention of vitamin B_3_ decreased during ultrasound application, except for the process carried out at 40 °C. Vitamin B_3_ is chemically bonded to nucleotides, and as much as 70% of niacin may be biologically unavailable in raw foods [17,18]. The retention of vitamin B_3_ was higher at 40 °C (an average of 102% at 40 °C against an average of 62% at 10 °C). Ultrasound application increased the content of the free-form of vitamin B_3_ by 11% in the experiments carried out at 40 °C and 5000 W/L, providing the highest availability of vitamin B_3_. A similar trend was also observed for ultrasound-assisted air-drying of apples, where a significant release of vitamin B_3_ was attained at 45 and 60 °C [9]. The analysis of perturbation of factors (Table 1) showed that the primary factor influencing the changes in vitamin B_3_ was the temperature (*p* < 0.01). Ultrasound power density presented a higher significance for vitamin B_3_ (*p* < 0.01) than for vitamin B_2_. The retention of vitamin B_3_ could be correlated with Equation (2) (*R*^2^ = 0.96).

(3) $$\mathrm{Retention}\;\mathrm{of}\;\mathrm{Vit}\;B_{3}\;\left(\%\right)=62.463-0.808T+0.037T^{2}-0.001P$$

The vitamin B_5_ content displayed a trend similar to the one presented by vitamin B_3_. The retention of vitamin B_5_ decreased during ultrasound application, except for the process carried out at 40 °C (Figure 1c). In fruits, this vitamin (pantothenic acid) exists mainly in its free-form [19] and, thus, more prone to degradation than other B vitamins. Vitamin B_5_ has good stability in food, but several works reported its loss during processing of legumes, cereals, beef [8] and apples [9]. The results of the analysis of perturbation of factors (Table 1) indicated that temperature was the most critical factor affecting vitamin B_5_ retention (*p* < 0.01), while the ultrasonic power density had very little statistical significance. The retention of vitamin B5 could be correlated with Equation (3) (*R*^2^ = 0.98).

(4) $${\mathrm{Retention}\;\mathrm{of}\;\mathrm{Vit}\;B}_{5}\;\left(\%\right)=97.248-3.524T+0.096T^{2}-0.008P$$

The kinetics of vitamins B showed that the retention of vitamins B decreased during the first 5 min of ultrasound application and increased slightly after 10 or 15 min, for low and room temperature. At these temperatures, the cavitation effect increases forming more free-radicals and transferring more energy to the food matrix molecules, which can lead to degradation of certain molecules, like the free-form of vitamins B. As the processes continues, the bonded vitamins tend to be detached from their apoenzymes, generating more free-form vitamins, leading to a slight increase in their content.

Vitamin C content did not change significantly during ultrasonic processing (Figure 2). This is an important finding because acerola is a major source of vitamin C. A reduction in vitamin C content would be extremely troublesome because the major appeal of acerola juice is due to its high vitamin C content. The changes in vitamin C were not statistically significant (at a 95% level of confidence), although numerically, its content increased slightly (6% at 10 °C and 4% at 40 °C) [20,21]. Equation (4) correlates the retention of vitamin C with the operating conditions (*R*^2^ = 0.99).

(5) $$\mathrm{Retention}\;\mathrm{of}\;\mathrm{Vit}\;C\;\left(\%\right)=96.474-8.00T+18.63T^{2}-5.33P-11.37P^{2}-8.50TP$$

Pro-vitamin A was affected by temperature, ultrasonic power density and processing time (Figure 3a,b). The increase in the free-form of pro-vitamin A was possible applying ultrasound at 10 °C and 40 °C. The increase in pro-vitamin A content was probably caused by cavitation, which may be responsible for the release of pro-vitamin A from pulp cell membranes or their lipoproteins. The analysis of the perturbation of factors (Table 3) showed that the main factor affecting the retention of pro-vitamin A was the temperature (*p* < 0.01). The retention of pro-vitamin A was correlated with the process variables through Equation (5) (*R*^2^ = 0.97). The kinetics of pro-vitamin A presented the same trend as the B vitamins. Its content decreased during the first 5 min of ultrasound application and increased slightly after 10 min, for low and room temperature.

(6) $$\mathrm{Retention}\;\mathrm{of}\;\mathrm{Vit}\;A\;\left(\%\right)=131.304-6.480T+0.137T^{2}-0.002P$$

A significant increase (average of 280%) in vitamin E was observed when the process was carried out at 10 °C and with short processing times (<10 min) (Figure 3c,d). Increasing the temperature caused a reduction in the retention of vitamin E. For example, the retention of vitamin E decreased from 88% to 45% when the process temperature was increased from 25 °C to 40 °C.

The radical scavenging behavior of vitamin E played a role in its degradation since ultrasound application induces the production of free radicals that react with vitamin E. This vitamin was also affected by the thermal effect. For instance, the retention of vitamin E dropped to 5% at high temperature and high ultrasonic power density (40 °C, 5000 W/L and 15 min of processing). Even at 10 °C, where an increase in vitamin E was observed, prolonged exposure to ultrasound imparted in a drop of over 50% in the vitamin E content (considering the peak concentration).

Analysis of the perturbation of factors (Table 2) showed that two factors influenced the changes in vitamin E content: the temperature (*p* < 0.01) and the ultrasound power density (*p* < 0.05). The retention of vitamin E was correlated with the process variables by Equation (6) (*R*^2^ = 0.97).

(7) $$\mathrm{Retention}\;\mathrm{of}\;\mathrm{Vit}\;E\;\left(\%\right)=470.390-21.656T+0.256T^{2}-0.001P$$

### 3.2. Cloud Stability

**Figure 4:**
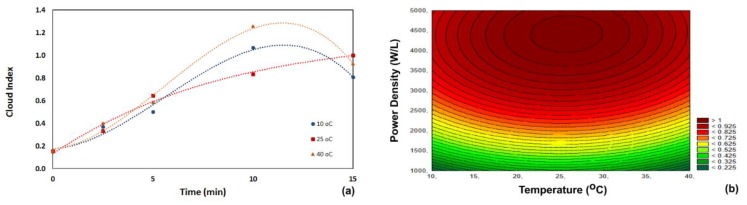
Influence of temperature and processing time on the cloud index of acerola juice. (**a**) Process carried out at 5000 W/L; (**b**) process carried out for 15 min.

The cloud value of the untreated acerola juice was 0.156 ± 0.002. Sonication increased the homogeneity of the juice. The visual aspect of the juice improved, and no phase separation was observed (common in untreated acerola juice). The cloud value in the sonicated acerola juice increased with increasing processing time (Figure 4). The juices with a cloud value higher than 1.0 remained as a single-phase liquid for three weeks (when our test stopped). All sonicated acerola juice samples reached a cloud value of 1.0 (after 10 min of sonication at 10 and 40 °C and after 15 min of sonication at 25 °C). Overexposure to sonication reduced the cloud index, as observed between 10 min and 15 min of sonication at 10 and 40 °C.

This improved homogeneity of sonicated juices was also observed for cantaloupe melon juice and orange juice [6,22]. Analysis of the perturbation of factors showed that the main factor influencing the changes in cloud value content was the ultrasonic power density (*p* < 0.05) (Table 4). The cloud value was correlated with the process variables by Equation (7) (*R*^2^ = 0.99).

(8) $$\mathrm{Cloud}\;\mathrm{value}=-0.497+0.034T-0.001T^{2}+0.001P$$

## 4. Conclusions

Ultrasonic processing was able to increase the content of the free-form of vitamins A, B_2_, B_3_, B_5_ and E, releasing the vitamin from its bond to the membrane, protein or apoenzyme, at specific operating conditions. The increase in vitamins A, B_2_, B_3_ and B_5_ was favored at high temperature, while the retention of vitamin E was favored at low temperature. The retention of vitamin C was high at all operating conditions.

The stability of the juice, measured by the cloud value, increased under all conditions that were studied. Phase separation was significantly reduced. Thus, ultrasonic processing was beneficial to the stability and nutritional quality of acerola juice.

## Figures and Tables

**Table 1:** Experimental design applied in the study on the effects of sonication in acerola juice.

Run	Power Density (W/L)	Temperature (°C)	Time (min)
1	1000	10	2.5, 5, 10, 15
2	1000	25	2.5, 5, 10, 15
3	1000	40	2.5, 5, 10, 15
4	3000	10	2.5, 5, 10, 15
5 (C)	3000	25	2.5, 5, 10, 15
6	3000	40	2.5, 5, 10, 15
7	5000	10	2.5, 5, 10, 15
8	5000	25	2.5, 5, 10, 15
9	5000	40	2.5, 5, 10, 15
10 (C)	3000	25	2.5, 5, 10, 15

**Table 2:** Analysis of the perturbation of factors for vitamins B_2_, B_3_ and B_5_ in acerola juice subjected to ultrasound processing.

Factor	Effect	Standard Error	*p*
Vitamin B_2_			
Mean	77.91	1.26	0
Temperature	19.62	2	0.0002
Temperature ^2^	27.77	3.09	0.0003
Power Density	6.45	2.01	0.0237
Power Density ^2^	−1.84	3.09	0.578
Temp × Power Density	−1.2	2.46	0.6455
Vitamin B_3_			
Mean	74.04	2.22	0
Temperature	39.92	3.53	0.0001
Temperature ^2^	17.05	5.43	0.0257
Power Density	15.3	3.52	0.0075
Power Density ^2^	2.67	5.43	0.6437
Temp × Power Density	6.34	3.32	0.2026
Vitamin B_5_			
Mean	58.76	1.77	0
Temperature	45.96	2.83	0.0001
Temperature ^2^	44.15	4.34	0.0002
Power Density	−4.2	2.82	0.1965
Power Density ^2^	6.19	4.34	0.2132
Temp × Power Density	4.62	3.45	0.2389

**Table 3:** Analysis of the perturbation of factors for vitamins C, pro-vitamin A and vitamin E in acerola juice subjected to ultrasound processing.

Factor	Effect	Standard Error	*p*
Vitamin C			
Mean	96.67	3.26	0
Temperature	−8.54	5.18	0.1602
Temperature ^2^	18.15	7.97	0.0718
Power Density	−5.35	5.18	0.349
Power Density ^2^	−11.55	7.97	0.2072
Temp × Power Density	−8.54	6.34	0.2363
Pro-vitamin A			
Mean	55.87	5.23	0.0001
Temperature	6.88	8.33	0.4468
Temperature ^2^	90.74	12.82	0.0009
Power Density	11.13	8.34	0.2393
Power Density ^2^	3.59	12.84	0.7907
Temp × Power Density	−12.59	10.2	0.272
Vitamin E			
Mean	95.48	10.94	0.0003
Temperature	−207.21	17.42	0.0001
Temperature ^2^	113.59	26.8	0.0082
Power Density	−47.68	17.42	0.0409
Power Density ^2^	−37.93	26.81	0.2162
Temp × Power Density	38.18	21.33	0.1335

**Table 4:** Analysis of the perturbation of factors for the cloud value in acerola juice subjected to ultrasound processing.

Factor	Effect	Standard Error	*p*
Mean	0.902	0.07	0.001
Temperature	0.01	0.076	0.9022
Temperature ^2^	−0.289	0.132	0.1157
Power Density	0.651	0.077	0.0034
Power Density ^2^	−0.437	0.131	0.0454
Temp × Power Density	0.025	0.093	0.8098

## References

[1] Piyasena P., Mohareb E., McKellar R.C. (2003). Inactivation of microbes using ultrasound: A review. Int. J. Food Microbiol..

[2] Gamboa-Santos J., Montilla A., Soria A.C., Villamiel M. (2012). Effects of conventional and ultrasound blanching on enzyme inactivation and carbohydrate content of carrots. Eur. Food. Res. Technol..

[3] Jang J.-H., Moon K.-D. (2011). Inhibition of polyphenol oxidase and peroxidase activities on fresh-cut apple by simultaneous treatment of ultrasound and ascorbic acid. Food Chem..

[4] Lee H., Zhou B., Feng H., Martin S.E. (2009). Effect of pH on inactivation of escherichia coli K12 by sonication, manosonication, thermosonication, and manothermosonication. J. Food Sci..

[5] Costa M.G.M., Fonteles T.V., Jesus A.L.T., Almeida F.D.L., Miranda M.R.A., Fernandes F.A.N., Rodrigues S. (2011). High-Intensity Ultrasound Processing of Pineapple Juice. Food Bioprocess Technol..

[6] Fonteles T.V., Costa M.G.M., de Jesus A.L.T., Miranda M.R.A., Fernandes F.A.N., Rodrigues S. (2012). Power ultrasound processing of cantaloupe melon juice: Effects on quality parameters. Food Res. Int..

[7] Anese M., Mirolo G., Beraldo P., Lippe G. (2013). Effect of ultrasound treatments of tomato in vitro bioaccessibility. Food Chem..

[8] Ball G.F.M. (2006). Vitamins in Foods: Analysis, Bioavailability, and Stability.

[9] Fernandes F.A.N., Rodrigues S., Cárcel J.A., García-Pérez J.V. (2015). Ultrasound-Assisted Air-Drying of Apple (*Malus domestica* L.) and Its Effects on the Vitamin of the Dried Product. Food Bioprocess Technol..

[10] Fernandes F.A.N., Rodrigues S., García-Pérez J.V., Cárcel J.A. (2016). Effects of Ultrasound-Assisted Air Drying on Vitamins and Carotenoids of Cherry Tomatoes. Dry Technol..

[11] USDA (2015). USDA National Nutrient Database for Standard Reference. Release 28.

[12] Jedlicka A., Klimes J. (2005). Determination of Water- and Fat-Soluble Vitamins in Different Matrices Using High-Performance Liquid Chromatography. Chem. Pap..

[13] Rizzolo A., Polesello S. (1992). Chromatographic determination of vitamins in foods. J. Chromatogr..

[14] Selimović A., Salkić M., Selimović A. (2011). Direct Spectrophotometric Determination of l—Ascorbic acid in Pharmaceutical Preparations using Sodium Oxalate as a Stabilizer. Int. J. Basic Appl. Sci..

[15] Versteeg C., Rombouts F.M., Spaansen C.H., Pilnik W. (1980). Thermostability and orange juice cloud destabilizing properties of multiple pectinesterases from orange. J. Food Sci..

[16] Mason T.J., Peters D. (2002). Practical Sonochemistry: Power Ultrasound Uses and Applications.

[17] Wall J.S., Carpenter K.J. (1988). Variation in availability of niacin in grain products. Food Technol..

[18] Ghosh H.P., Sarkar P.K., Guha B.C. (1963). Distribution of the bound form of nicotinic acid in natural materials. J. Nutr..

[19] Plesovsky-Vig N., Shils M.E., Olson J.A., Shike M., Ross A.C. (1999). Pantothenic acid. Modern Nutrition in Health and Disease.

[20] Fernandes F.A.N., Oliveira V.S., Gomes W.F., Rodrigues S. (2016). Degradation kinetics of vitamin E during ultrasound application and the adjustment in avocado puree by tocopherol acetate addition. LWT Food Sci. Technol..

[21] Di Mascio P., Kaiser S., Sies H. (1989). Lycopene as the Most Efficient Biological Carotenoid Singlet Oxygen Quencher. Arch. Biochem. Biophys..

[22] Tiwari B.K., Muthukumarappan K., O’Donnell C.P., Cullen P.J. (2009). Inactivation kinetics of pectin methylesterase and cloud retention in sonicated orange juice. Innov. Food Sci. Emerg. Technol..

